# Genetic characterisation of the influenza viruses circulating in Bulgaria during the 2019–2020 winter season

**DOI:** 10.1007/s11262-021-01853-w

**Published:** 2021-06-22

**Authors:** Neli Korsun, Ivelina Trifonova, Silvia Voleva, Iliyana Grigorova, Svetla Angelova

**Affiliations:** Department of Virology, National Laboratory “Influenza and ARI”, National Centre of Infectious and Parasitic Diseases, Sofia, Bulgaria

**Keywords:** Influenza virus, Genetic characterisation, Amino acid substitution

## Abstract

Influenza viruses have a high potential for genetic changes. The *objectives* of this study were to analyse influenza virus circulation in Bulgaria during the 2019/2020 season, to perform a phylogenetic and molecular analyses of the haemagglutinin (*HA*) and neuraminidase (*NA*) sequences of representative influenza strains, and to identify amino acid substitutions compared to the current vaccine strains. Seasonal influenza viruses A(H3N2), A(H1N1)pdm09 and B/Victoria-lineage were detected using a real-time RT-PCR in 323 (23.3%), 149 (10.7%) and 138 (9.9%) out of 1387 patient samples studied, respectively. The *HA* genes of A(H3N2) viruses analysed belonged to clades 3C.3a (21 strains) and 3C.2a (5 strains): subclades 3C.2a1b + T131K, 3C.2a1b + T135K-B and 3C.2a1b + T135K-A. The clade 3C.3a and subclade 3C.2a1b viruses carried 5 and 14–17 substitutions in *HA,* as well as 3 and 9 substitutions in *NA*, respectively, in comparison with the A/Kansas/14/2017 vaccine virus, including some substitutions in the *HA* antigenic sites A, B, C and E. All 21 A(H1N1)pdm09 viruses sequenced fell into 6B.1A5A subclade. Amino acid sequence analysis revealed the presence of 7–11 substitutions in *HA*, compared to the A/Brisbane/02/2018 vaccine virus, three of which occurred in antigenic site Sb, along with 6–9 changes at positions in *NA*. All 10 B/Victoria-lineage viruses sequenced belonged to clade 1A with a triple deletion in *HA1* (genetic group 1A(Δ3)B) and carried 7 and 3 substitutions in *HA* and *NA*, respectively, with respect to the B/Colorado/06/2017 vaccine virus. The results of this study confirm the rapid evolution of influenza viruses and the need for continuous antigenic and genetic surveillance.

**Supplementary Information:**

The online version contains supplementary material available at 10.1007/s11262-021-01853-w.

## Introduction

Influenza is one of the most common infectious illnesses in humans that affects annually 5–10% of the adult population and 20–30% of children causing a huge number of outpatient visits, hospitalisations and fatal cases, thus imposing a substantial public health burden [1]. Since 2009, two subtypes of influenza A viruses, A(H1N1)pdm09 and A(H3N2), and two genetic lineages of B viruses, B/Victoria and B/Yamagata, have been circulating during seasonal epidemics. There are temporal and geographical variations in their prevalence, clinical activity, as well as in the antigenic and genetic characteristics of the circulating strains.

Influenza viruses belong to the *Orthomyxoviridae* family and have a segmented, negative-sense, single-stranded RNA genome, each segment of which encodes at least one protein [2]. The surface glycoproteins haemagglutinin (*HA*) and neuraminidase (*NA*) form discrete spikes and play an important part in a viral life cycle. *HA* mediates the binding of the virus to the sialic acids as cellular receptors and the entry of the viral ribonucleoprotein into the cell cytoplasm by fusing of viral envelope and cell membrane. *NA* destroys sialic acid residues on the cell surface and provides the release of newly formed influenza viruses from the infected cells. *HA* is the primary target for virus-neutralising antibodies and together with *NA* is a key component of influenza vaccines.

Vaccination is currently the best way to reduce the influenza morbidity and mortality, but the effectiveness of influenza vaccines is often suboptimal due to the highly variable nature of influenza viruses and their ability to evade the existing humoral immunity. Understanding the molecular mechanisms by which influenza viruses avoid the immunity induced by a prior natural infection or vaccination is crucial for the development of effective prevention and control strategies. Due to the absence of a proofreading capability of the viral RNA polymerase complex and the high replication rate of influenza viruses, point mutations occur constantly in the viral genome. Mutations which enable the virus to overcome host immune protection, are accumulated in *HA* and, to a lesser extent, in *NA* as a result of immune selection pressure, a process known as antigenic drift [3]. Mutant viruses with altered antigenic characteristics are capable of causing reinfections in immune individuals and repeated epidemics in the general population. It has been found that a limited number of amino acid substitutions at the antibody-binding sites around the receptor-binding pocket can result in escape recognition of neutralising antibodies. With the help of escape mutants, five epitopes of neutralising antibodies, known as antigenic sites, have been identified in the globular heads of the *HAs* of both A(H1N1) (Ca1, Ca2, Cb, Sa, Sb) and A(H3N2) (A–E) viruses [4, 5]. Influenza type B viruses contain four major immunodominant epitopes in the *HA1* subunit comprising the 120-loop, 150-loop, 160-loop, and 190-helix [6]. In addition to mutations, glycosylation of *HA* and *NA* (attachment of an oligosaccharide to the sequon N X S/T, where X may represent any amino acid except P) is another important mechanism used by viruses to evade the antibody-mediated immunity. Recent studies have suggested that the attached glycan chains shield antigenically significant regions reducing access of antibodies and protect the enzymatic sites of *NA* [7]. The frequent emergence of new genetic variants of influenza viruses that are rapidly replacing previously circulating strains, necessitates regular updates in the influenza vaccines composition in order to match epidemic strains.

Continuous monitoring of circulating influenza viruses is needed to identify new genetic variants of influenza viruses with the potential to avoid host immune defence, with increased virulence, transmissibility or reduced susceptibility to antiviral drugs. The main *objectives* of this study were to analyse the influenza virus circulation in Bulgaria during the 2019/2020 season and to determine the genetic and molecular characteristics of the detected viruses as compared to the current vaccine strains.

## Materials and methods

### Study population and specimen collection

**Fig. 1:**
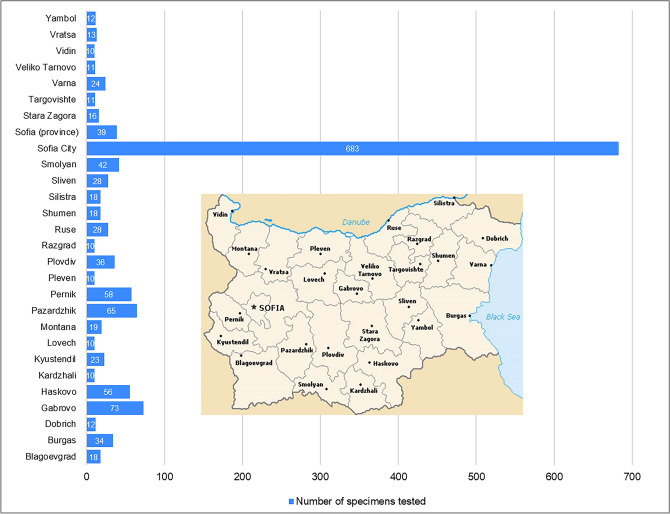
Number of specimens taken for influenza virus testing in all 28 regions of Bulgaria, 2019/2020 season

From October 2019 to March 2020, patients experiencing acute respiratory infection (ARI) from all 28 regions of the country were enrolled in the national influenza surveillance programme (Fig. 1). The diagnosis of each patient was determined by their attending physician based on standard clinical criteria. Nasopharyngeal specimens from the enrolled patients were collected using polyester collection swabs during the visit to the general practitioner or within the first 24 h of admission. The containers containing a nasopharyngeal swab placed into 2 ml of virus transport medium, were stored at 4 °C for up to 72 h and shipped to the National Laboratory “Influenza and ARI”, which is recognised by the World Health Organization (WHO) as a National Influenza Centre. Specimens were processed immediately or stored at − 80 °C before testing.

### Extraction of viral nucleic acids and real-time RT-PCR

Viral RNAs were extracted automatically from nasopharyngeal specimens using a commercial ExiPrep Dx Viral DNA/RNA kit and ExiPrep 16DX equipment (BioNeer, Korea) in accordance with the manufacturer’s instructions. Clinical specimens were screened for influenza viruses by a real-time RT-PCR method using a SuperScript III Platinum® One-Step qRT-PCR System (Invitrogen, USA). Primers, probes and positive controls were provided by the International Reagent Resource, USA. Amplification was performed with a CFX96 thermal cycler (Bio-Rad) in accordance with the protocol recommended by the Centers for Disease Control and Prevention (CDC), Atlanta, USA (reverse transcription at 50 °C for 30 min, Taq activation at 95 °C for 2 min, followed by 45 cycles of denaturation at 95 °C for 15 s and annealing at 55 °C for 30 s) [8].

### Viral isolation and antigenic characterisation

PCR-positive clinical specimens with a high viral load (*C*_*t*_ value < 28) were selected and inoculated onto cultures of Madin Darby canine kidney (MDCK) and MDCK-SIAT1 (a cell line expressing increased levels of sialyl-α2,6-galactose moieties [9]). Cultures were incubated at 35 °C in an atmosphere of 5% CO_2_ and observed daily for 7 days for visual cytopathic effect. The presence of virus in culture was confirmed by haemagglutination assay following standard protocols using a 1% suspension of guinea pig red blood cells. Antigenic characterisation of influenza isolates was performed by the haemagglutination inhibition (HAI) assay following the WHO Manual, using vaccine viruses/antigens and their corresponding antisera kindly provided by the WHO Collaborating Centres (WHO-CCs) in London and CDC, Atlanta [10]. More detailed HAI assays of representative Bulgarian influenza isolates with panels of reference viruses and antisera were performed at the WHO-CCs in London and Atlanta. Viral isolates were considered as antigenically related to the vaccine virus if they showed no more than a fourfold reduced HAI titre with antiserum raised against the vaccine virus, as compared to the homologous titre. A reduction of at least eightfold in the HAI titres was considered a signal of antigenic difference.

### Genetic characterisation

The sequencing of influenza viruses detected in Bulgaria during the 2019/2020 season was performed at the WHO-CCs in London and Atlanta. Consensus sequences have been deposited in the EpiFlu database of the Global Initiative on Sharing All Influenza Data (GISAID) [11]. For phylogenetic analyses, sequences of study viruses, reference viruses with known genetic group identities determined by the WHO-CCs [12], vaccine strains, and viruses representing different countries of Europe during the 2019/2020 season, were retrieved from the EpiFlu database of GISAID (Supplementary Table). Multiple alignments for *HA* and *NA* sequences were performed using the MUSCLE algorithm embedded in the MEGA, version 6.06 software [13]. The best-fit nucleotide substitution models for phylogenetic analysis of *HA* (Hasegawa-Kishino-Yano model with a gamma distribution, HKY + G) and *NA* (Tamura 3-parameter model with gamma distribution, T92 + G) were determined using MEGA 6.06. Phylogenetic trees were constructed using Maximum Likelihood method. *HA* and *NA* nucleotide sequences of the Bulgarian influenza viruses analysed in this study are available in GISAID under the identifiers presented in the Supplementary Table.

### Deduced amino acid sequence analysis and prediction of N-glycosylation motifs

Deduced amino acid sequences were generated by translating nucleotide sequences with the standard genetic code using MEGA 6.06 software. The amino acid sequence identities were calculated using FluServer [14]. The presence of putative *N*-linked glycosylation sites, N X S/T (sequon), where X can be any amino acid except proline, was identified using the NetNGlyc 1.0 Server [15].

### Statistics

The detection rates of the individual viruses were compared using Chi-square or Fisher’s exact tests for categorical variables. *P* values < 0.05 were considered statistically significant.

## Results

**Fig. 2:**
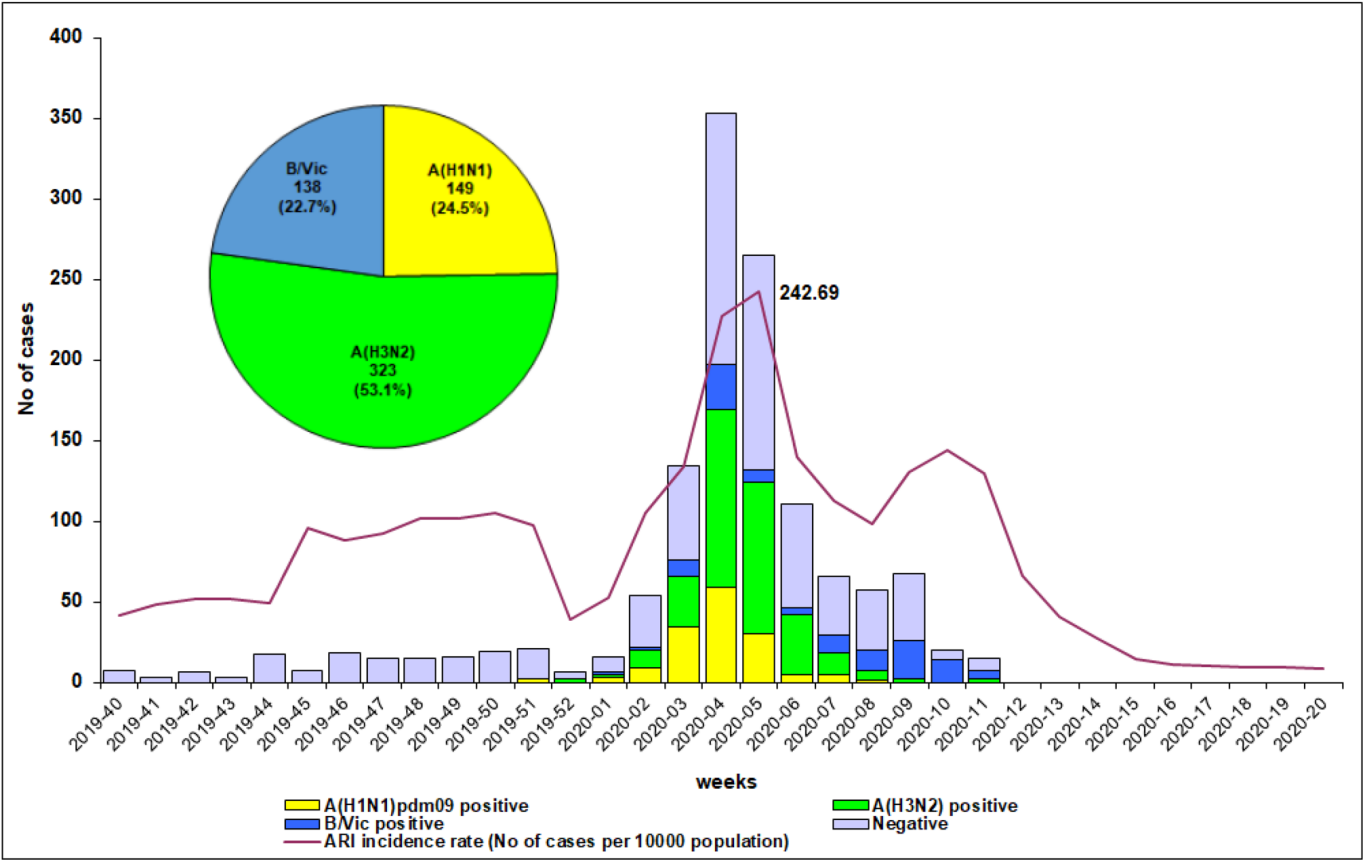
Weekly distribution of patients tested for influenza viruses in Bulgaria, season 2019/2020

The 2019/2020 influenza epidemic lasted for 6 weeks (weeks 2–8 of 2020) and peaked at week 5. A second, small peak in morbidity due to B/Victoria-lineage viruses was registered at week 10 of 2020 (Fig. 2).

### Patient characteristics

A total of 1387 patients presenting with ARI were tested for influenza viruses. About 23.3% (323/1387) of these patients attended outpatient healthcare centres, 76.7% (1064/1387) were hospitalised patients, of which 27 were treated in intensive care units (ICUs). The patients’ ages ranged from 13 days to 88 years; 676 (48.7%) of the participants were male. Most clinical samples (683, 49.2%) were obtained from patients living in the capital Sofia. The number of samples tested from other regions ranged from 10 to 73 (Fig. 1).

### Influenza virus detection

Influenza viruses were identified in 610 (44%) study subjects: 138 (42.7%) outpatients and 472 (44.4%) inpatients. Influenza type A and type B represented 77.4% (472/610) and 22.6% (138/610) of all detected influenza viruses, respectively. Of the influenza A cases, 323 (68.4%) were subtyped as A(H3N2) and 149 (31.6%) were subtyped as A(H1N1)pdm09. All 138 (22.6%) type B viruses were determined to be B/Victoria-lineage. The proportions of influenza A(H3N2), A(H1N1)pdm09 and B/Victoria-lineage virus infections among outpatients were 24.5% (79/323), 9.9% (32/323), and 8.4% (27/323), respectively, while among the inpatients these proportions were as follows: 22.9% (244/1064), 11% (117/1064), and 10.4% (111/1064), respectively, without statistically significant differences. The percentage of detected A(H1N1)pdm09 viruses in the capital Sofia was similar to that in other regions of the country (10% vs 11.5%), while the percentage of detected A(H3N2) viruses was higher in the regions outside Sofia (26.3% vs 20.2%, *P* < 0.05), and the percentage of detected B/Victoria-lineage viruses was higher in Sofia city (13% vs 6.7%, *P* < 0.05).

The first influenza detection, an A(H1N1)pdm09 virus, occurred in week 51/2019, and the last, an A(H3N2) virus, occured in week 11/2020. Increased circulation of influenza B/Victoria-lineage viruses was seen at weeks 8–10 of 2020 (Fig. 2). After week 11/2020, influenza virus testing was discontinued due to overloading of the laboratory with SARS-CoV-2 testing.

### Phylogenetic and antigenic characterisation

A total of 57 influenza viruses detected in 13 clinical specimens and 70 cell-culture isolates, were sequenced at WHO-CCs in London and Atlanta and deposited in the EpiFlu database of GISAID. Phylogenetic analysis was performed to determine the affiliation of Bulgarian strains to the genetic groups of globally circulating influenza viruses. The antigenic characterisation using HAI assays was aimed to determine the antigenic similarity of the Bulgarian isolates with the current vaccine strains.

**Fig. 3:**
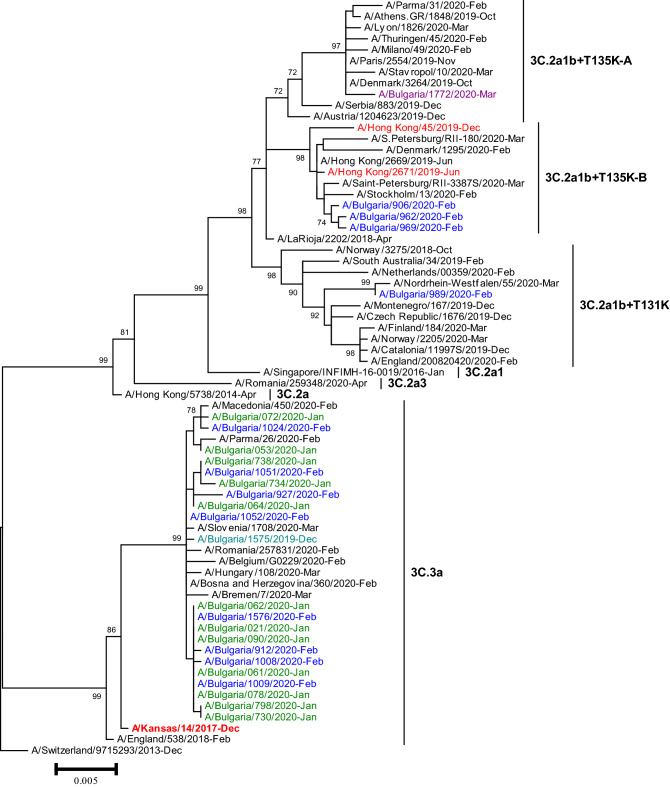
Phylogenetic analysis of the *HA* nucleotide sequences from influenza A(H3N2) viruses detected in Bulgaria during the 2019/2020 season. The tree is rooted at A/Switzerland/9715293/2013. Vaccine viruses A/Kansas/14/2017 (egg-based, 2019–2020 NH influenza season), A/Hong Kong/2671/2019 (egg-based, 2020–2021 NH and 2021 SH influenza seasons) and A/Hong Kong/45/2019 (cell/recombinant-based, 2020–2021 NH and 2021 SH influenza seasons) are indicated in red. Bulgarian viruses detected from December 2019 to March 2020 are indicated in teal, green, blue and purple, respectively

The *HA* genes of 26 A(H3N2) viruses sequenced fell into genetic clade 3C.3a (21/26, 80.8%) and clade 3C.2a, subclades 3C.2a1b + T131K (1/26, 3.8%), 3C.2a1b + T135K-B (3/26, 11.5%) and 3C.2a1b + T135K-A (1/26, 3.8%) (Fig. 3). The eight clade 3C.3a viruses and one subclade 3C.2a1b + T135K-A virus analysed by HAI assay were inhibited by the post-infection ferret antisera raised against the egg-propagated northern hemisphere (NH) 2019/2020 vaccine virus A/Kansas/14/2017 (clade 3C.3a) at titres 2- to 4-fold lower than the homologous titre of the antiserum. The three subclade 3C.2a1b + T135K-B viruses analysed by HAI assay exhibited > fourfold titre reductions compared to the vaccine virus and were antigenically distinct.

**Fig. 4:**
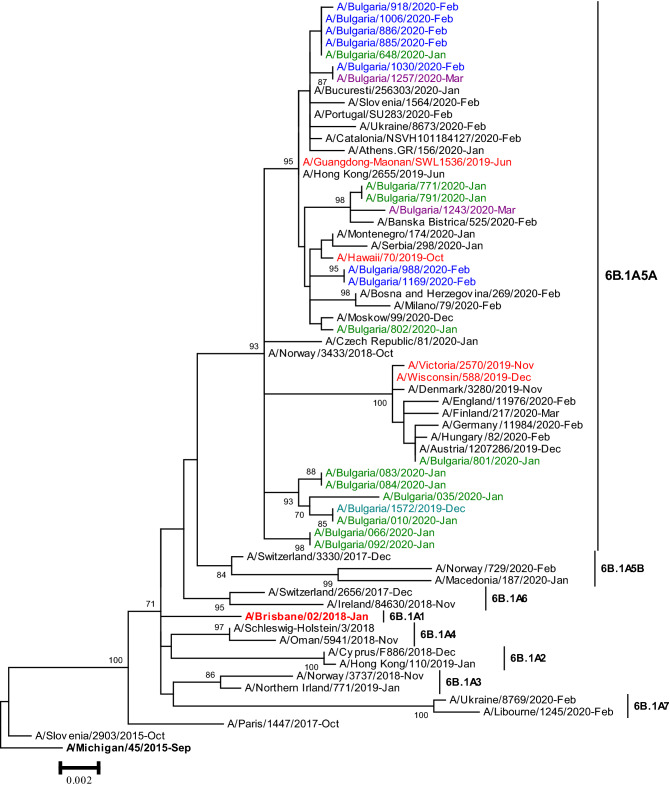
Phylogenetic analysis of the *HA* nucleotide sequences from influenza A(H1N1)pdm09 viruses detected in Bulgaria during the 2019/2020 season. The tree is rooted at A/Michigan/45/2015. Vaccine viruses A/Brisbane/02/2018 (egg-based, 2019–2020 NH influenza season), A/Guangdong-Maonan/SWL1536/2019 (egg-based, 2020–2021 NH influenza season), A/Hawaii/70/2019 (cell/recombinant-based, 2020–2021 NH influenza season), A/Victoria/2570/2019 (egg-based, 2021 SH influenza season), and A/Wisconsin/588/2019 (cell/recombinant-based, 2021 SH influenza season), are indicated in red. Bulgarian viruses detected from December 2019 to March 2020 are indicated in teal, green, blue and purple, respectively

Phylogenetic analysis of the *HA* genes of all 21 A(H1N1)pdm09 viruses sequenced showed that they clustered within the subclade 6B.1A5A (Fig. 4). Ferret antisera raised against egg-propagated vaccine virus A/Brisbane/02/2018 inhibited the eleven antigenically characterised A(H1N1)pdm09 viruses at titres equal to, or 2- to 4-fold lower than the homologous titre of the antiserum.

**Fig. 5:**
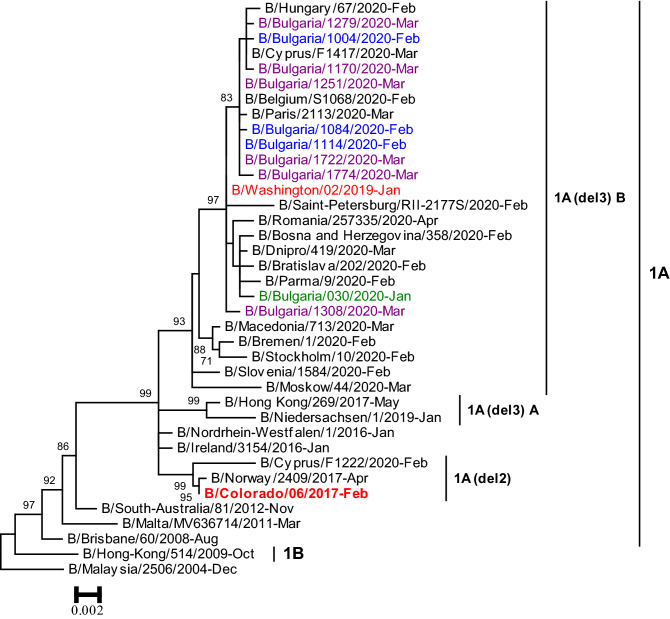
Phylogenetic analysis of the *HA* nucleotide sequences from influenza B/Victoria-lineage viruses detected in Bulgaria during the 2019/2020 season. The tree is rooted at B/Malaysia/2506/2004. Vaccine viruses B/Colorado/06/2017 (2019–2020 NH influenza season) and B/Washington/02/2019 (2020–2021 NH and 2021 SH influenza season) are indicated in red. Bulgarian viruses detected from January to March 2020 are indicated in green, blue and purple, respectively

All the 10 B/Victoria-lineage viruses belonged to *HA* clade 1A with a three amino acid deletion in *HA1* (positions 162–164) (Fig. 5). The nine viruses analysed by HAI assay were poorly recognised by antisera raised against egg-propagated vaccine virus, B/Colorado/06/2017, which contains a two amino acid deletion in *HA* (positions 162 and 163).

### HA and NA protein sequence analysis

*HA* and *NA* protein sequences of influenza viruses circulating in Bulgaria during the 2019/2020 season were compared to those of egg-propagated vaccine strains in order to identify substitutions that could affect vaccine effectiveness.

#### A(H3N2)

The comparison of the *HA* amino acid sequences of 26 Bulgarian A(H3N2) strains with the NH 2019/2020 vaccine strain, A/Kansas/14/2017 (clade 3C.3a), revealed a similarity ranged from 98.763 to 99.293% for clade 3C.3a viruses and from 95.583 to 96.643% for subclade 3C.2a1b viruses [14]. *HAs* of Bulgarian clade 3C.3a viruses differed from that of vaccine virus with substitutions N190D (all 21 strains), K264E (*n* = 5) and K276R (*n* = 11) in the *HA1* domain; and M17L (*n* = 21) and A201V (*n* = 21) in the *HA2* domain. Viruses in subclades 3C.2a1b had more changes compared to the vaccine virus: E50K (*n* = 3), E62G (*n* = 5), N91S (*n* = 5), K92R (*n* = 5), N121K (*n* = 5), T135K (*n* = 4), S137F (*n* = 3), K144S (*n* = 5), S159Y (*n* = 5), K160T (*n* = 5) resulting in the acquisition of an *N*-linked glycosylation sequon, N171K (*n* = 5), N190D (*n* = 4) and R326K (*n* = 5) in *HA1*; I77V (*n* = 5), M149I (*n* = 5) and G155E (*n* = 4) in *HA2*. Nine substitutions in subclade 3C.2a1b viruses were located at the antigenic sites: T135K, S137F and K144S—at site A; S159Y, K160T and N190D—at site B; E50K—at site C; E62G and N91S—at site E. Substitution N190D was located on 190-helix (188–194), which is part of antigenic site B, as well as the receptor-binding site (RBS); S144K was very close to the RBS. Thirteen potential *N*-glycosylation sites in *HA* (*HA1* positions 8, 22, 38, 45, 63, 122, 126, 133, 158, 165, 246, and 285, and *HA2* position 154) were identified. Six of them were located at the antigenic epitopes: position 63 (antigenic site E); positions 122, 126 and 133 (antigenic site A); and positions 158 and 246 (antigenic site B).

All the Bulgarian clade 3C.3a viruses shared the *NA* amino acid substitutions I57M, N161S and P386S when they were compared with the sequence of A/Kansas/14/2017 strain. The five viruses in subclades 3C.2a1b possessed several *NA* amino acid substitutions: R75K, P126L, I140L, A149V, H155Y, K220N, V303I, S315R and T329S. None of the *NA* sequences analysed contained substitutions in the catalytic site residues (R118, D151, R152, R224, E276, R292, R371, and Y406) that directly contact the sialic acid, and in the framework residues (E119, R165, W178, S179, D198, I222, E227, H274, E277, N294, and E425) that support the catalytic site [16]. Eight potential *N*-linked glycosylation sequons (at positions 61, 70, 86, 146, 200, 234, 245 and 367) were identified, two of which (146 and 367) were located around the enzyme active site [17].

#### A(H1N1)pdm09

*HA* amino acid sequence identity of the 21 viruses studied ranged from 97.350 to 98.587% compared to the vaccine virus, A/Brisbane/02/2018 [14]. All the A(H1N1)pdm09 *HA* sequences contained 3 amino acid changes in *HA1*, N129D, T185I and N260D, which defined the 6B.1A5A subclade and several additional substitutions: G45R/K, R223Q, A282P and V298I. Thirteen strains fell in subgroup carrying *HA1* substitutions D187A and Q189E [12].

Three of the identified amino acid substitutions, T185I, D187A and Q189E, were located at antigenic site Sb. The strain A/Bulgaria/801/2020 fell in subgroup 6B.1A5A+156K as A/Victoria/2570/2019, the vaccine virus for the 2021 southern hemisphere (SH) influenza season, and carried several additional substitutions, two of which were located at antigenic site Sa [18]. Nine conserved potential *N*-glycosylation motifs were encoded by the *HA* genes of the 21 Bulgarian A(H1N1)pdm09 viruses: at *HA1* positions 10/11, 23, 87, 162, 276, 287 and *HA2* positions 154 and 213. The *N*-glycosylation motif at position 162 was located in the antigenic site Sa.

The 20 available *NA* sequences from Bulgarian A(H1N1)pdm09 viruses differed from that of A/Brisbane/02/2018 at several amino acid positions: T13I (all 20 strains), I40T (*n* = 7), Q51K (*n* = 20), S52N (*n* = 13) (with the loss of an *N*-glycosylation sequon), T72N (*n* = 7) (with the gain of an *N*-glycosylation sequon), F74S (*n* = 20), I389K (*n* = 20), D416N (*n* = 20) and T452I (*n* = 20). Two amino acid substitutions were located at the *NA* antigenic sites including the residues 83–143, 156–190, 252–303, 330, 332, 340–345, 368, 370, 387–395, 400, 431–435, and 448–468 [19]. *NA* catalytic site residues (118R, 119E, 151D, 152R, 179W, 223I, 225R, 277E, 293R, 368R, and 402Y) and their supporting framework residues (156R, 180S, 228E, 247S, 278E, and 295N) were highly conserved [20]. Eight potential *N*-glycosylation motifs at positions 42, 50, 58, 63, 68, 88, 146 and 235 were found.

#### B/Victoria

All the 10 sequenced Bulgarian B/Victoria-lineage viruses belonged to clade 1A with a triple deletion (Δ162–164) in *HA1*. Compared to the current vaccine virus, B/Colorado/06/2017, there was a *HA* amino acid similarity between 98.110 and 98.797% [14]. All Bulgarian strains fell in group encoding the K136E substitution in *HA1* (genetic group 1A(Δ3)B). Several other amino acid substitutions were also identified in *HA*: G129D (*n* = 10), G133R (*n* = 10), V180I (*n* = 10), T197N (*n* = 10) resulting in the gain of a potential *N*-glycosylation site, N233K (*n* = 8) resulting in the loss of a potential *N*-glycosylation motif in *HA1*, and K151R (*n* = 10) in *HA2*. Three amino acid variations were found in the antigenic 120-loop area (positions 116–137); T197N was identified in the 190-helix area (positions 194–202). Twelve potential *N*-linked glycosylation sites were identified at *HA1* positions 25, 59, 145, 166, 197, 233, 304 and 333, and *HA2* positions 145, 171, 184 and 216. *N*-linked glycosylation sites 145, 166 and 197 were located at the antigenic sites: 150-loop (positions 141–150), 160-loop (positions 162–167) and 190-helix, respectively [21].

The sequenced Bulgarian B/Victoria-lineage viruses carried three *NA* amino acid substitutions: S283G (*n* = 6), Q371K (*n* = 7) and A395T (*n* = 7). The eight catalytic residues (R116, D149, R150, R223, E275, R292, R374, and Y409) and their 11 framework residues (E117, R154, W177, S178, D197, I221, E226, H273, E276, N293, E428) within *NA* were conserved among all 7 viruses studied [22]. Four putative *N*-glycosylation motifs at positions 56, 64, 144 and 284 were identified.

## Discussion

In the present study, the circulation pattern and genetic characteristics of influenza viruses detected in Bulgaria during the 2019/2020 season were examined. Influenza A(H3N2) viruses prevailed (53.1%), and A(H1N1)pdm09 and B/Victoria-lineage viruses circulated in similar proportions (24.5% and 22.7%, respectively). No B/Yamagata-lineage viruses were identified. Cumulative data for the WHO European region showed that most viruses detected (73%) were type A with a slight predominance (56%) of A(H1N1)pdm09 viruses over A(H3N2) viruses (44%), and 27% were type B, with 98% of them determined to be B/Victoria-lineage [23].

In Bulgaria, influenza A viruses were predominant for most seasons after the 2009/2010 pandemic, except for the 2012/2013 and 2017/2018 seasons. Strong dominance of influenza A(H3N2) viruses was observed in the 2011/2012, 2014/2015 and 2016/2017 seasons, and increased circulation of A(H1N1)pdm09 viruses was seen during the 2010/2011, 2013/2014, and 2015/2016 epidemics [24, 25].

Among the seasonal influenza viruses, A(H3N2) have been characterised by the highest rate of evolution and significant genetic diversity [26–28]. Since 2009, seven genetic groups and multiple clades/subclades based on the *HA* gene have been defined within this subtype. In 2014, two genetically and antigenically divergent genetic clades, 3C.2a and 3C.3a, emerged and continued to circulate widely worldwide in the following seasons [29]. Viruses in clade 3C.2a rapidly became predominant in most geographic areas including in Bulgaria [25, 30]. Over this period, 3C.2a viruses diverged into several subclades: 3C.2a1 (3C.2a1a and 3C.2a1b), 3C.2a2, 3C.2a3, and 3C.2a4 [16]. Unlike the previous four seasons, during the 2019/2020 influenza epidemic in Bulgaria, the majority (80.8%) circulating A(H3N2) viruses fell into the genetic clade 3C.3a, and the remainder (19.2%) belonged to subclade 3C.2a1b, which included three subdivisions and was not represented in the vaccine. The clade 3C.3a viruses were antigenically and genetically close to the vaccine virus, while those of subclade 3C.2a1b and its subdivisions differed significantly due to the presence of a great number of amino acid mismatches, 6–9 of which were within the *HA* antigenic sites A, B, C and E (Table 1). Antigenic sites B and A, located on the top of *HA1* around the RBS are main targets of human neutralising antibodies. It has been found that from 1968 to 2003, the major antigenic changes have been caused mainly by single amino acid substitutions which occur at only seven positions in *HA* (145 in site A and 155, 156, 158, 159, 189, 193 in site B) near the RBS [31]. In our study, substitution S159Y was found in one of these key positions. *HAs* of A(H3N2) viruses have been characterised by a high degree of glycosylation, which probably facilitates immune evasion. Previous studies showed that the number of *HA* glycosylation sequons in A(H3N2) viruses increases in the years following their introduction into the human population. While the pandemic A(H3N2) strain Hong Kong/1/68 harboured two N-glycosylation sites at positions 81 and 165 on the head of *HA*, the most recent representatives contained 7 additional N-glycosylation sites on the *HA* globular head and 5 on the stem region [16]. Six potential *N*-glycosylation motifs were identified at the antigenic regions of Bulgarian A(H3N2) viruses. Attachment of oligosaccharide chains to the antigenic sites of *HA* may alter the antigenicity and virulence of influenza viruses [32]. The glycan at position 158 is located immediately adjacent to the amino acid residues at positions 156, 158 and 159 that are responsible for major antigenic cluster transitions.

**Table 1:** Number of positions with amino acid changes compared to the vaccine viruses and number of potential N-linked glycosylation motifs in HA and NA of influenza viruses circulating in Bulgaria during the 2019/2020 season

Influenza viruses	Vaccine strains	Number of positions with amino acid changes compared to the vaccine viruses			*N*-glycosylation motifs	
		*HA*		*NA*	*HA*	*NA*
		Total number	Positions at antigenic regions			
A(H1N1)pdm09	A/Brisbane/02/2018	7	1	6	9	8
A(H1N1)pdm09 + D187A and Q189E		9	3	9	9	8
A(H1N1)pdm09 + N156K		11	3	9	9	8
A(H3N2)/clade 3C.3a	A/Kansas/14/2017	5	–	3	13	8
A(H3N2)/subclade 3C.2a1b		14–17	6–9	9	13	8
B/Victoria-lineage	B/Colorado/06/2017	7	3	3	12	4

Since the 2009/2010 pandemic, the A(H1N1)pdm09 virus has evolved continuously and has undergone significant diversification in eight major genetic groups and several clades/subclades based on the *HA* gene. The appearance of the recent clade 6B.1 viruses at the beginning of the NH 2015/2016 season was accompanied by many severe and fatal influenza cases in a number of countries, including Bulgaria [33, 34]. The vast majority of recently globally circulating viruses formed a subclade designated 6B.1A5A and all sequenced Bulgarian A(H1N1)pdm09 viruses belonged to this subclade. A total of 7–11 amino acid variations (three located at antigenic site Sb and two at site Sa) were identified in *HA* and 6–9 in *NA* compared to the vaccine virus, A/Brisbane/2/2018 (Table 1). Attachment of glycan at position 162 of the antigenic site Sa is likely to affect antibody recognition. Despite these genetic changes, subclade 6B.1A5A viruses remained antigenically similar to the vaccine virus as assessed by the use of post-infection ferret antisera raised against the vaccine virus [12]. However, due to the increasing number of viruses A(H1N1)pdm09+D187A and Q189E, which were antigenically distinguishable from the vaccine virus by panels of post-vaccination human sera from the 2019–2020 NH influenza season, A/Guangdong-Maonan/SWL1536/2019 virus was recommended as a vaccine component for the 2020/2021 NH influenza season [35].

In Bulgaria, all type B viruses detected in 2019/2020 belonged to the Victoria-lineage. Previously, an increased circulation of B/Victoria-lineage viruses was observed in the country in the 2015–2016 season [33]. Recently circulating viruses with a double (Δ162–163) or a triple deletion (Δ162–164) in *HA1* appeared for the first time during the 2016/2017 season [36]. Seven amino acid substitutions in *HA1*, four of which were located in the antigenic 120-loop (*n* = 3) and 190-helix (*n* = 1), and 5 substitutions in *NA* were identified in Bulgarian strains with respect to the current vaccine virus (Table 1). According to previous studies, the 120-loop exhibits a highest degree of variations [37]. The 190-helix is a part of the RBS. Amino acid substitutions within these regions could potentially alter antigenicity and virulence [22, 38, 39]. This statement was confirmed by the results of antigen characterisation, which showed that the Bulgarian B/Victoria-lineage viruses were antigenically distinct from the contemporary vaccine component.

In conclusion, co-circulation of seasonal influenza viruses A(H3N2), A(H1N1)pdm09 and B/Victoria-lineage was observed during the 2019/2020 season in Bulgaria. The results of this study showed the emergence of new genetic variants, some of which had alterations in antigenicity compared to the current vaccine strains. The study confirms the rapid evolution of influenza viruses and the need for continuous antigenic and genetic surveillance.

## Supplementary Information

Below is the link to the electronic supplementary material.

Supplementary file1 (DOCX 24 KB)

## Data Availability

The datasets analysed during the current study are available in the GISAID database (https://www.gisaid.org/).
