# Supplementary material for: Genetic characterisation of the influenza viruses circulating in Bulgaria during the 2019–2020 winter season
Source: Virus Genes. 2021 Jun 22;57(5):401–12. doi: 10.1007/s11262-021-01853-w (PMC8217981; doi:10.1007/s11262-021-01853-w)
Supplement: Supplementary file 1 — Supplementary file1 (DOCX 24 KB) [file 11262_2021_1853_MOESM1_ESM.docx]

**Supplementary Table**. GISAID virus/sequence identification/accession numbers used in this study

| **A(H1N1)pdm09 strains** | **Accession number** | **A(H3N2) strains** | **Accession number** |
| --- | --- | --- | --- |
| A/Brisbane/02/2028  A/Michigan/45/2015 | EPI_ISL_237793  EPI_ISL_200780 | A/Kansas/14/2017  A/Singapore/INFIMH-16-0019/2016 | EPI_ISL_312833  EPI_ISL_344420 |
| A/Slovenia/2903/2015  A/Guangdong Maonan/SWL1536/2019  A/Victoria/2570/2019  A/Wisconsin/588/2019  A/Paris/1447/2017  A/Hong Kong/110/2019  A/Cyprus/F886/2018  A/Oman/5941/2018  A/Schleswig-Holstein/3/2018  A/Ireland/84630/2018  A/Switzerland/2656/2017  A/Macedonia/187/2020  A/Norway/729/2020  A/Switzerland/3330/2017  A/Norway/3433/2018  A/Hong Kong/2655/2019  A/Denmark/3280/2019  A/Hawaii/70/2019  A/Norway/3737/2018  A/Northern Ireland/771/2019  A/Ukraine/8769/2020  A/Libourne/1245/2010  A/Germany/11984/2020  A/England/11976/2020  A/Finland/217/2020  A/Portugal/SU283/2020  A/Catalonia/NSVH101184127/2020  A/Bosna and Herzegovina/269/2020  A/Banska Bistrica/525/2020  A/Austria/1207286/2019  A/Moskow/99/2020  A/Czech Republic/81/2020  A/Athens.GR/156/2020  A/Hungary/82/2020  A/Milano/79/2020  A/Montenegro/174/2020  A/Bucuresti/256303/2020  A/Serbia/298/2020  A/Slovenia/1564/2020  A/Ukraine/8673/2020  A/Bulgaria/1572/2019  A/Bulgaria/010/2020  A/Bulgaria/035/2020  A/Bulgaria/066/2020  A/Bulgaria/083/2020  A/Bulgaria/084/2020  A/Bulgaria/092/2020  A/Bulgaria/1030/2020  A/Bulgaria/1243/2020  A/Bulgaria/648/2020  A/Bulgaria/771/2020  A/Bulgaria/791/2020  A/Bulgaria/801/2020  A/Bulgaria/802/2020  A/Bulgaria/885/2020  A/Bulgaria/886/2020  A/Bulgaria/918/2020  A/Bulgaria/988/2020  A/Bulgaria/1006/2020  A/Bulgaria/1169/2020  A/Bulgaria/1257/2020  **B/Victoria-lineage strains**  B/Brisbane/60/2008  B/Malaysia/2506/2004  B/Hong-Kong/514/2009  B/Malta/MV636714/2011  B/South-Australia/81/2012  B/Nordrhein-Westfalen/1/2016  B/Ireland/3154/2016  B/Norway/2409/2017  B/Colorado/06/2017  B/Cyprus/F1222/2020  B/Niedersachsen/1/2019  B/Washington/02/2019  B/Moskow/44/2020  B/Macedonia/713/2020  B/Bremen/1/2020  B/Stockholm/10/2020  B/Slovenia/1584  B/Belgium/S1068/2020  B/Paris/2113/2020  B/Cyprus/F1417/2020  B/Hungary/67/2020  B/Saint Petersburg/RII-2177S/2020  B/Romania/257335/2020  B/Bosna and Herzegovina/358/2020  B/Dnipro/419/2020  B/Parma/9/2020  B/Bratislava/202/2020 | EPI_ISL_237558  EPI_ISL_419003  EPI_ISL_548964  EPI_ISL_404527  EPI_ISL_291668  EPI_ISL_338461  EPI_ISL_342128  EPI_ISL_332853  EPI_ISL_347329  EPI_ISL_338060  EPI_ISL_294119  EPI_ISL_409906  EPI_ISL_477594  EPI_ISL_294121  EPI_ISL_332840  EPI_ISL_377239  EPI_ISL_400732  EPI_ISL_400916  EPI_ISL_347404  EPI_ISL_338534  EPI_ISL_508599  EPI_ISL_498345  EPI_ISL_463776  EPI_ISL_453768  EPI_ISL_467724  EPI_ISL_455540  EPI_ISL_463731  EPI_ISL_462107  EPI_ISL_462102  EPI_ISL_409861  EPI_ISL_527250  EPI_ISL_411170  EPI_ISL_407681  EPI_ISL_527226  EPI_ISL_486734  EPI_ISL_416272  EPI_ISL_411167  EPI_ISL_409928  EPI_ISL_429942  EPI_ISL_527274  EPI_ISL_409738  EPI_ISL_409474  EPI_ISL_407700  EPI_ISL_409714  EPI_ISL_409870  EPI_ISL_411168  EPI_ISL_411169  EPI_ISL_429886  EPI_ISL_429887  EPI_ISL_429888  EPI_ISL_429889  EPI_ISL_429890  EPI_ISL_429892  EPI_ISL_429893  EPI_ISL_429894  EPI_ISL_429895  EPI_ISL_429896  EPI_ISL_429897  EPI_ISL_527196  EPI_ISL_527201  EPI_ISL_527203  **Accession number**  EPI_ISL_28264  EPI_ISL_29398  EPI_ISL_70224  EPI_ISL_99941  EPI_ISL_145989  EPI_ISL_208671  EPI_ISL_208680  EPI_ISL_268030  EPI_ISL_276408  EPI_ISL_486776  EPI_ISL_348804  EPI_ISL_347843  EPI_ISL_527308  EPI_ISL_500766  EPI_ISL_429946  EPI_ISL_420887  EPI_ISL_429956  EPI_ISL_477329  EPI_ISL_498446  EPI_ISL_486782  EPI_ISL_527292  EPI_ISL_419102  EPI_ISL_459985  EPI_ISL_527280  EPI_ISL_527219  EPI_ISL_486800  EPI_ISL_462139 | A/Switzerland/9715293/2013  A/England/538/2018  A/Hong Kong/5738/2014  A/Romania/259348/2020  A/Norway/3275/2018  A/South Australia/34/2019  A/LaRioja/2202/2018  A/Denmark/3264/2019  A/Paris/2554/2019  A/Hong Kong/45/2019  A/Hong Kong/2669/2019  A/Hong Kong/2671/2019  A/Hungary/108/2020  A/Bremen/7/2020  A/Bosna_and_Herzegovina/360/2020  A/Parma/26/2020  A/Macedonia/450/2020  A/Slovenia/1708/2020  A/Romania/257831/2020  A/Belgium/G0229/2020  A/Nordrhein-Westfalen/55/2020  A/Serbia/883/2019  A/Czech Republic/1676/2019  A/Montenegro/167/2019  A/Catalonia/11997S/2019  A/Milano/49/2020  A/England/200820420/2020  A/Norway/2205/2020  A/Finland/184/2020  A/Netherlands/00359/2020  A/Parma/31/2020  A/Lyon/1826/2020  A/Austria/1204623/2019  A/Stavropol/10/2020  A/Athens.GR/1848/2019  A/Thuringen/45/2020  A/S.Petersburg/RII-180/2020  A/Saint-Petersburg/RII-3387S/2020  A/Stockholm/13/2020  A/Denmark/1295/2020  A/Bulgaria/1575/2019  A/Bulgaria/021/2020  A/Bulgaria/078/2020  A/Bulgaria/090/2020  A/Bulgaria/072/2020  A/Bulgaria/064/2020  A/Bulgaria/053/2020  A/Bulgaria/061/2020  A/Bulgaria/062/2020  A/Bulgaria/798/2020  A/Bulgaria/1008/2020  A/Bulgaria/1009/2020  A/Bulgaria/1024/2020  A/Bulgaria/1052/2020  A/Bulgaria/1772/2020  A/Bulgaria/730/2020  A/Bulgaria/734/2020  A/Bulgaria/738/2020  A/Bulgaria/906/2020  A/Bulgaria/912/2020  A/Bulgaria/927/2020  A/Bulgaria/962/2020  A/Bulgaria/969/2020  A/Bulgaria/989/2020  A/Bulgaria/1576/2020  A/Bulgaria/1051/2020  **B/Victoria-lineage strains**  B/Bulgaria/1004/2020  B/Bulgaria/1114/2020  B/Bulgaria/1251/2020  B/Bulgaria/1279/2020  B/Bulgaria/1308/2020  B/Bulgaria/1722/2020  B/Bulgaria/1170/2020  B/Bulgaria/1774/2020  B/Bulgaria/030/2020  B/Bulgaria/1084/2020 | EPI_ISL_164719  EPI_ISL_312041  EPI_ISL_165629  EPI_ISL_516793  EPI_ISL_332813  EPI_ISL_400538  EPI_ISL_314925  EPI_ISL_406276  EPI_ISL_407511  EPI_ISL_347938  EPI_ISL_377300  EPI_ISL_377302  EPI_ISL_527221  EPI_ISL_434393  EPI_ISL_498296  EPI_ISL_486739  EPI_ISL_477582  EPI_ISL_434438  EPI_ISL_515567  EPI_ISL_477420  EPI_ISL_481457  EPI_ISL_397315  EPI_ISL_416202  EPI_ISL_410007  EPI_ISL_416185  EPI_ISL_486726  EPI_ISL_487077  EPI_ISL_500740  EPI_ISL_485741  EPI_ISL_415732  EPI_ISL_486744  EPI_ISL_498357  EPI_ISL_409943  EPI_ISL_477610  EPI_ISL_407444  EPI_ISL_434446  EPI_ISL_477602  EPI_ISL_471284  EPI_ISL_413665  EPI_ISL_477517  EPI_ISL_407466  EPI_ISL_407463  EPI_ISL_407464  EPI_ISL_407465  EPI_ISL_409368  EPI_ISL_409369  EPI_ISL_409370  EPI_ISL_409954  EPI_ISL_409955  EPI_ISL_429891  EPI_ISL_434394  EPI_ISL_434395  EPI_ISL_434396  EPI_ISL_434397  EPI_ISL_434398  EPI_ISL_434399  EPI_ISL_434400  EPI_ISL_434401  EPI_ISL_434402  EPI_ISL_434403  EPI_ISL_434404  EPI_ISL_434405  EPI_ISL_434406  EPI_ISL_434407  EPI_ISL_491799  EPI_ISL_527199  **Accession number**  EPI_ISL_429947  EPI_ISL_429949  EPI_ISL_429950  EPI_ISL_429951  EPI_ISL_429952  EPI_ISL_429953  EPI_ISL_429954  EPI_ISL_429967  EPI_ISL_462624  EPI_ISL_477337 |
